# Mutilation on gill filaments of *Piaractus brachypomus* (Characiformes: Serrasalmidae) caused by *Braga patagonica* (Crustacea: Cymothoidae), in the Brazilian Amazon

**DOI:** 10.1590/S1984-29612025068

**Published:** 2025-12-08

**Authors:** Marcos Sidney Brito Oliveira, Marcos Tavares-Dias

**Affiliations:** 1 Universidade do Estado do Amapá – UEAP, Macapá, AP, Brasil; 2 Universidade Federal do Amapá – UNIFAP, Programa de Pós-graduação em Biodiversidade Tropical – PPGBio, Macapá, AP, Brasil; 3 Empresa Brasileira de Pesquisa Agropecuária – Embrapa Amapá, Macapá, AP, Brasil

**Keywords:** Amazon, aquaculture, damage, Isopoda, pirapitinga, Amazônia, aquicultura, danos, Isopoda, pirapitinga

## Abstract

Fish aquaculture is one of the main food production sectors for the growing global human population and is currently one of the most profitable production activities. However, parasitic infestations have emerged as a significant factor influencing fish aquaculture production, negatively impacting the profitability of this food production activity. This study aimed to report the mutilation of pirapitinga *Piaractus brachypomus* gill filaments caused by a Cymothoidae parasite. In April 2022, *Piaractus brachypomus* were examined at a fish farm in Macapá, state of Amapá, Brazil. The host fish were analyzed, and the parasite found was preserved in 70% ethyl alcohol for taxonomic identification. Of the 31 fish examined, one had its gills infested with a crustacean parasite identified as *Braga patagonica*. A female parasite caused macroscopic mutilation of the filaments on the four right gill arches of *P. brachypomus* and severe damage at the attachment site. This is the first record of *B. patagonica* infestation in *P. brachypomus*. The fish farming water supply allowed the isopod ectoparasite to pass from wild fish to the pond, where it consequently found favorable conditions in the host fish.

## Resumo

A piscicultura é um dos principais setores produtores de alimentos para a população humana global, e também uma das atividades de produção mais lucrativas atualmente. No entanto, infestações parasitárias têm emergido como uma grande influência na produção de peixes, impactando negativamente a lucratividade dessa atividade econômica de produção de alimentos. O objetivo deste estudo foi relatar mutilação em filamentos branquiais de *Piaractus brachypomus* (pirapitinga), causada por um parasito Cymothoidae. *Piaractus brachypomus* foram examinadas em abril de 2022 em uma piscicultura da cidade de Macapá, no estado do Amapá, Brasil. Os peixes hospedeiros foram analisados, e os parasitos encontrados foram preservados em álcool etílico a 70% para identificação taxonômica. Dos 31 peixes examinados, um estava com as brânquias infestadas por um crustáceo parasito identificado como *Braga patagonica*. Uma fêmea de *B. patagonica* causou mutilação macroscópica nos filamentos dos quatro arcos branquiais direitos de *P. brachypomus*. Este crustáceo ectoparasito foi observado causando danos graves neste sítio de fixação em *P. brachypomus*. Este é o primeiro registro de infestação por *B. patagonica* em *P. brachypomus*. O abastecimento de água na piscicultura possibilitou a passagem deste ectoparasito isópode de peixes selvagens para o viveiro, que consequentemente encontrou condições favoráveis neste peixe hospedeiro.

## Introduction

Fish aquaculture is one of the main food production activities for the growing global human population and is one of the world's most profitable production sectors. However, several factors, including parasitism, can negatively affect this intensive production, thus impacting the profitability of this important industry (Horton & Okamura, 2001; FAO, 2024).

In aquaculture, fish can become infested with various ectoparasites, including species of isopod crustaceans from the family Cymothoidae Leach, 1814 (Horton & Okamura, 2001; Sahoo et al., 2022). South America has the greatest diversity of Cymothoidae species (Tavares-Dias et al., 2014). These crustaceans belong to the order Isopoda Latreille, 1817, and comprise approximately 386 species distributed in 46 genera (Ahyong et al., 2011; WoRMS, 2024).

Cymothoid isopods are ectoparasites that infest the tegument, fins, mouth, and gills of the host fish. They can cause severe pathology in their hosts, negatively affecting body growth and production in fish aquaculture (Brusca, 1978; Horton & Okamura, 2001, 2003; Tavares-Dias et al., 2014; Ali & Aboyadak, 2018; Abdallah & Hamouda, 2022; Sahoo et al., 2022; Alves et al., 2024). These crustaceans can easily be observed with the naked eye on host fish because of their large body size. For instance, the total length of female *Braga patagonica* Schioedte & Meinert, 1884 is approximately 24 mm, whereas that of males is approximately 21 mm (Tavares-Dias et al., 2014). In addition, *B. patagonica* is a freshwater parasitic isopod that lacks host specificity; it has been reported to infest different species of fish in the Brazilian Amazon and other regions due to its wide geographic distribution (Tavares-Dias et al., 2014; Alves et al., 2024), except *Piaractus brachypomus* Cuvier, 1818 (pirapitinga). The present study reports a case of gill filament mutilation in *P. brachypomus* caused by *B. patagonica* in a fish farm in Macapá, state of Amapá, Brazil.

## Material and Methods

**Figure 1:**
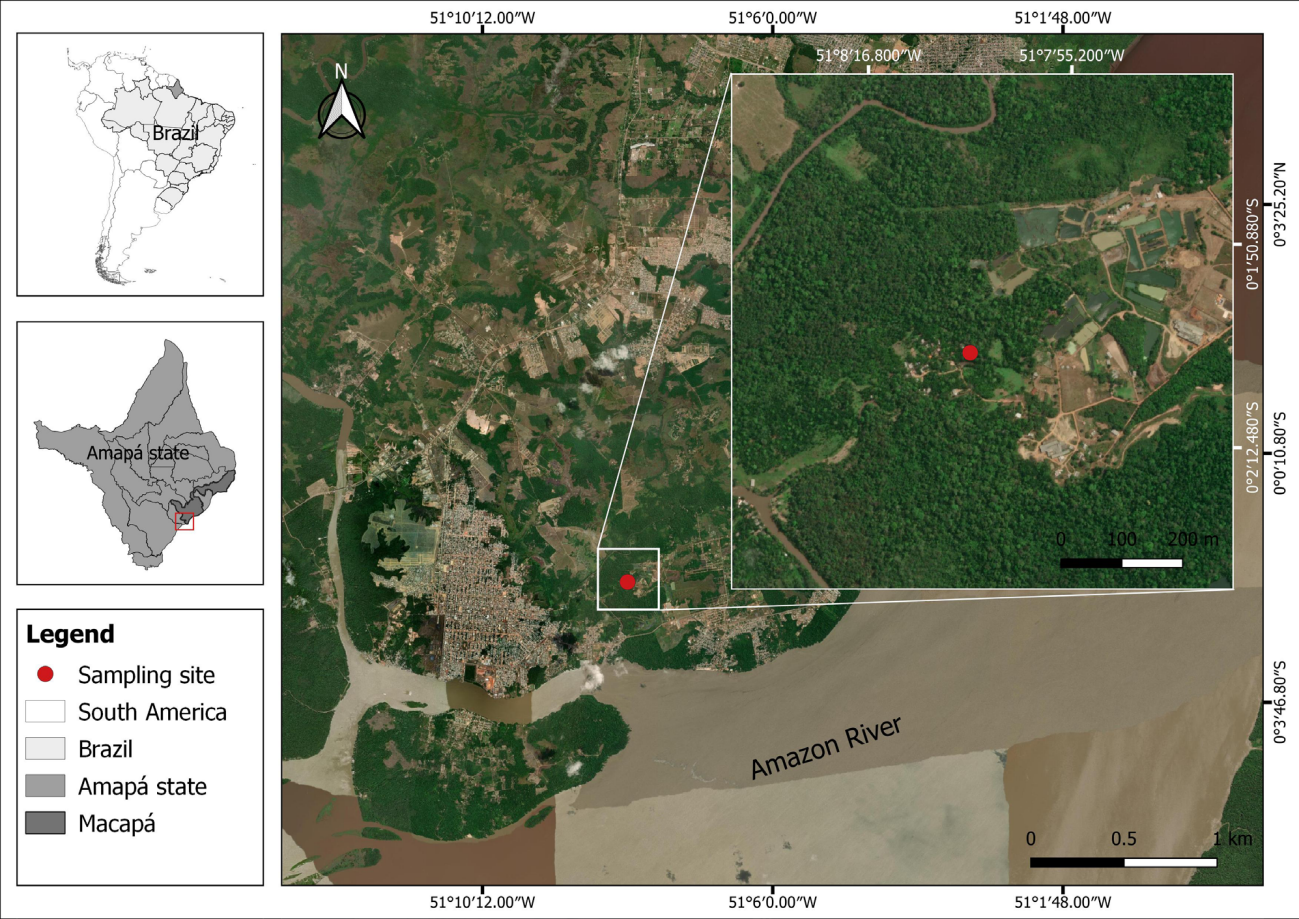
Geographic location of the fish farm for the collection of *Piaractus brachypomus*.

In April 2022, 31 specimens of *P. brachypomus* were collected from a fish farm (0°2'2.40"S 51°8'6.00"W) located in a rural area of the municipality of Macapá, state of Amapá, Brazil (Figure 1).

The fish farm consisted of two ponds (40 m × 20 m × 1.5 m) excavated in the ground, but only one pond was active. The ponds are supplied with water from the Fortaleza Stream, which is influenced by the waters of the Amazon River. Thus, due to the tides, water flows in and out of the ponds. Consequently, other fish species and parasites can enter the ponds through the water because the supply channels do not have any type of mesh to prevent aquatic organisms from entering.

The fish were collected using a trawl net and transferred to a box containing water and a constant supply of oxygen. Each fish was then transferred to a smaller box (40 L) containing 50 mg/L eugenol as an anesthetic. The fish were then weighed (g) and measured for total length (cm). Their oral cavities, tongues, and gills were examined to evaluate the presence of ectoparasitic crustaceans. The fish were then transferred to another box (40 L) with constant oxygenation until they fully recovered. After the analysis, the fish were returned to their respective ponds. The parasites were fixed in 70% ethyl alcohol for identification.

The total length (TL) and total width (TW) of the parasite were measured in millimeters (mm). For identification purposes, the appendages (antennae, pleopods, and uropods) were removed and transferred to a Petri dish containing 5% potassium hydroxide (Oliveira et al., 2021). The dish was then placed on a hot plate at 60 °C for approximately 20 minutes to clarify the sample (the time varies depending on the size of the appendages). Each appendage was then mounted between a slide and a coverslip in pure glycerin medium, and the preparation was sealed with paraffin (Oliveira et al., 2021). Morphological identification was performed according to Lemos de Castro (1959). The prevalence, mean intensity, and mean abundance of the parasite were calculated (Bush et al., 1997).

## Results

The examined *P. brachypomus* specimens measured 32.8 ± 2.2 cm in length and weighed 812.9 ± 197.5 g.

**Figure 2:**
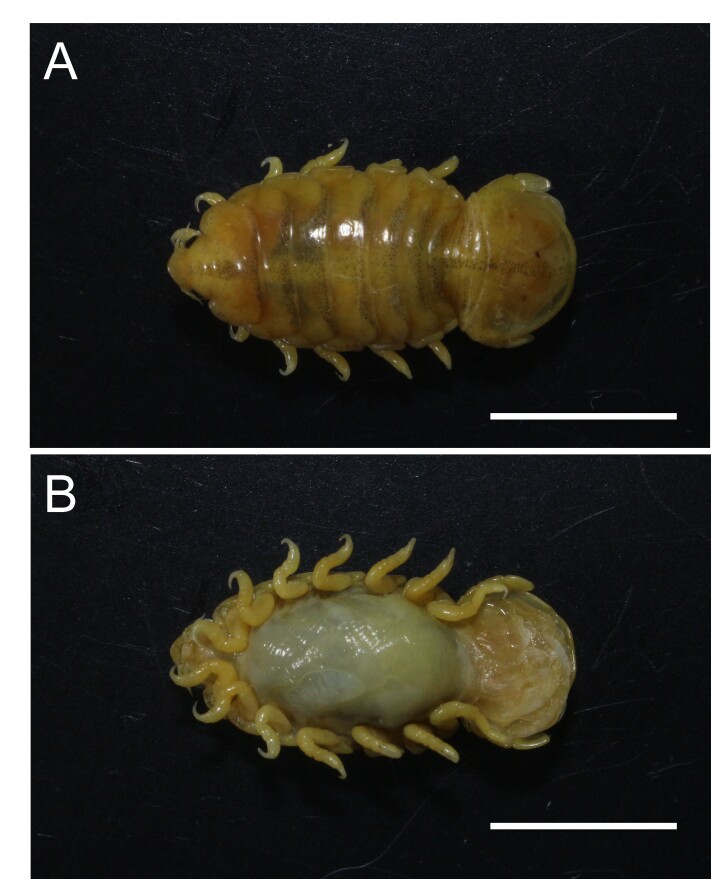
Specimen of *Braga patagonica* in dorsal (A) and ventral (B) view, collected from the gill of *Piaractus brachypomus*. Scale 5 mm.

The identified parasitic crustacean was a female *B. patagonica* (Figure 2) with a TL of 11.0 mm and a TW of 5.0 mm. The features to the diagnostic of the species were: Head triangular, projecting anteriorly, with a narrow, rounded front. Eyes small and rounded. Antennules and antennae short, composed of 8 and 9 articles, respectively. Thorax broad and rounded, widest at the 4th segment. The first thoracic segment is noticeably longer than the following segments. Pereiopods bear dactyli progressively longer and more robust from the 1st to the 6th pair, whereas the 7th pair is smaller. Total abdominal length is approximately equal to the length of the thorax, excluding the first thoracic segment. Telson well developed, with a width 1.5 times its length, and roughly 1.5 times longer than the combined length of the first abdominal segments, excluding the first. Posterior end truncated at the base and laterally, with a faint median carina. Uropods shorter than the telson, both rami elongated-oval, with the endopod distinctly shorter than the exopod.

**Figure 3:**
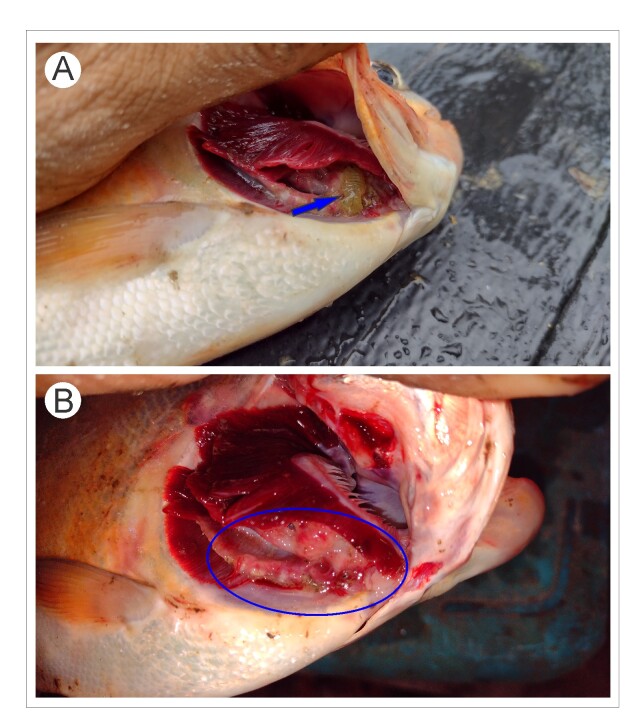
*Piaractus brachypomus* parasitized by *Braga patagonica*. (A) Arrow indicates the parasite specimen in the gill of the host fish; (B) Circle indicates the region of mutilation on the gill filaments caused by the parasite.

Of the 31 fish examined, only one had an individual of *B. patagonica* on its gills. The infestation levels were: prevalence = 3.2%, mean intensity = 1.0, and mean abundance = 0.03. Macroscopic mutilation of the filaments of the four right gill arches of *P. brachypomus* was observed at the site of *B. patagonica* attachment (Figure 3).

## Discussion

In Brazilian aquaculture, the main parasitic infestations in fish are typically caused by ectoparasites such as monogeneans, protozoans, and crustaceans. However, there are few reports on endoparasites, such as nematodes and acanthocephalans (Godoi et al., 2012; Silva et al., 2013; Dias et al., 2015a, b). For example, *Colossoma macropomum* Cuvier, 1816, tambatinga, and tambacu hybrids farmed in the state of Amapá, Brazil, have been infested with ectoparasites such as *Ichthyophthirius multifiliis* Fouquet, 1876, *Piscinoodinium pillulare* (Schäperclaus) Lom, 1981 (Protozoa), *Lernaea gamitanae* (Thatcher & Paredes, 1985) (Crustacea), and dactylogyridean species (Dias et al., 2015a, b). They are also infected with endoparasites, including *Procamallanus inopinatus* Travassos, Artigas & Pereira, 1928 (Nematoda) and *Neoechinorhynchus buttnerae* Golvan, 1956 (Acanthocephala) (Silva et al., 2013; Dias et al., 2015a, b). However, only farmed *C. macropomum* have been infested with *B. patagonica* due to infested *Hoplias malabaricus* Bloch, 1794 entering the fish farm's cultivation tanks through the water supply system without any control of invasive fish or parasites (Tavares-Dias et al., 2014; Dias et al., 2015a).

*Braga patagonica* is an ectoparasite that lacks host specificity that usually infests wild characiforms fish such as *H. malabaricus*; *Serrasalmus altispinis* Merckx, Jégu & Santos 2000; *Pygocentrus nattereri* Kner, 1858; *C. macropomum*; *Serrasalmus* sp.; *Serrasalmus rhombeus* (Linnaeus, 1766); *Mylossoma duriventre* Cuvier, 1818; *Brycon amazonicus* (Spix & Agassiz, 1829); *Hydrolycus scomberoides* Cuvier, 1819; *Acestrorhynchus falcatus* Bloch, 1794; *Curimata incompta* Vari, 1984; *Leporinus fasciatus* Bloch, 1794; and, in addition, Cichliformes *Chaetobranchus flavescens* Heckel, 1840 and *Chaetobranchopsis orbicularis* Steindachner, 1875; as well as a species of Siluriformes *Pimelodella humeralis* Slobodian, Akama & Dutra, 2017 (Tavares-Dias et al., 2014, 2015; Oliveira et al., 2017; Neves & Tavares-Dias, 2019; Virgilio et al., 2020). Therefore, there are few records of *B. patagonica* infesting farmed fish species. However, other Cymothoidae isopod species have been recorded in farmed fish. For example, *Cymothoa indica* Schioedte & Meinert, 1884 parasitizes *Lates calcarifer* Bloch, 1790 in India (Rajkumar et al., 2005), and *Ceratothoa oestroides* Risso, 1827 and *Nerocila orbignyi* Guérin, 1832 parasitize *Dicentrarchus labrax* Linnaeus, 1758 and *Sparus auratus* Linnaeus, 1758 in Turkey (Horton & Okamura, 2001).

There are no reports of *B. patagonica* infesting farmed or wild *P. brachypomus.* Therefore, this is the first record of this isopod for *P. brachypomus*. In addition, the low levels of infestation (prevalence, mean intensity, and mean abundance) of *B. patagonica* in *P. brachypomus* were similar to those reported for other wild fish species in different Amazonian tributaries (Tavares-Dias et al., 2014, 2015; Oliveira et al., 2017; Neves & Tavares-Dias, 2019; Virgilio et al., 2020). However, the gill infestation of *P. brachypomus* by *B. patagonica* occurred because the ponds were directly supplied with water from the Amazon River. Fish farming ponds are supplied by channels directly connected to the river, with no containment measures to prevent invasive fish and parasites from entering the ponds. The few reports of *B. patagonica* in farmed fish occur especially in the Amazon region, and this seems to be related to the lack of records of this information because culture ponds are frequently and directly supplied with water from rivers, channels, or streams. Consequently, we believe that fish farmers have often found this ectoparasite in farmed fish but have ignored the importance of documenting it due to a lack of interest or knowledge.

We observed mutilation of the gill filaments in *P. brachypomus* caused by *B. patagonica*, which is possibly due to this parasite's feeding method. Cymothoid species feed on the blood cells that irrigate fish gills (Horton & Okamura, 2003), but they also seem to feed on gill tissue. Interestingly, a particular case of *B. patagonica* parasitizing *C. macropomum* has been reported, in which the ectoparasite infested the dorsal fin, destroying the skin and scales and causing severe local inflammation (Tavares-Dias et al., 2014). Similarly, other Cymothoidae species also cause serious damage to the mouths, gills, and skin of the host fish. For example, *Cymothoa exigua* Schioedte & Meinert, 1884 destroyed the tongue of *Lutjanus guttatus* Steindachner, 1869 (Brusca & Gilligan, 1983). *Renocila thresherorum* Williams & Bunkley-Williams, 1980 provoked the destruction and massive loss of primary and secondary gill lamellae in *Mugil capito* Risso, 1827 (Ali & Aboyadak, 2018), and *Livoneca redmanii* Leach, 1818 caused destruction, pallor, and desquamation of gill filaments in *Dicentrarchus labrax* Linnaeus, 1758 (Abdallah & Hamouda, 2022).

## Conclusions

This first record of *B. patagonica* in *P. brachypomus* demonstrates the importance of controlling parasites in cultivation ponds to help prevent the introduction of invasive wild fish with parasites that may be transmitted to farmed fish. This is caused by an inadequate water supply in fish farming.
